# Hemispheric surgery in children: perisylvian technique

**DOI:** 10.3171/2024.4.FOCVID2451

**Published:** 2024-07-01

**Authors:** Helio Rubens Machado, Marcelo Volpon Santos

**Affiliations:** Department of Surgery and Anatomy, Division of Pediatric Neurosurgery, Center for Epilepsy Surgery in Children, Ribeirão Preto Medical School, University of São Paulo, Ribeirão Preto, São Paulo, Brazil

**Keywords:** epilepsy surgery, hemispherotomy, surgical technique

## Abstract

Hemispheric epilepsy is quite frequent in children, compared with adults, and encompasses pathological substrates as diverse as hemimegalencephaly, Rasmussen encephalitis, Sturge-Weber syndrome, and porencephaly, among others. These patients most often become pharmacoresistant and thus require surgical management. Although anatomical hemispherectomy is a possibility, the technique that is favored by most epilepsy surgery centers worldwide is functional hemispherotomy, which results in equivalent outcomes with fewer postoperative complications. Therefore, it is essential that pediatric epilepsy neurosurgeons become familiar with these techniques. The present video describes in detail all surgical aspects of the perisylvian hemispherotomy.

The video can be found here: https://stream.cadmore.media/r10.3171/2024.4.FOCVID2451

## Transcript

### 0:20 Introduction and Patient Preparation for Surgery.

This video is related to the technique of hemispherotomy in children, the perisylvian technique. Rasmussen in 1974 was the first neurosurgeon that proposed the term "disconnection," when he described his technique of functional hemispherectomy, meaning that his intention was to preserve the frontal and occipital poles but disconnect them.^1,2^ After the introduction of microsurgical techniques in the 1990s, Delalande was the first author to propose the term "hemispherotomy" in 1992, in the description of the vertical parasagittal hemispherotomy.^3^ He was soon followed by Villemure, who in 1993 described his technique of peri-insular hemispherotomy.^4,5^ The aim of this video is to describe his technique in detail as published in 1995 and a modification proposed by Shimizu and Maehara in 2000.^6^ After general anesthesia the patient’s head is immobilized and rotated contralateral to the side of the surgery. A craniotomy depicted in this picture is then performed. Neuronavigation is always mandatory.

### 1:44 Frontoparietal Opercular Window.

The first step is to delimitate the frontoparietal operculum, which will be resected. So, this is the exposure of the frontoparietal operculum and subpial dissection, and at the end we will resect this cortical portion of the frontoparietal cortex of the operculum. As you can see, the dissection is always subpial and the branches of the middle cerebral artery are completely exposed.

### 2:44 Resection of the Insula.

Then after the exposure of the insula we start the dissection of the branches of the middle cerebral artery; here you can see the M1 branches which are followed, coagulated, and cut. As you can see here, the M1 branches are totally exposed and coagulated. We follow then the M1 branches, and one by one we dissect, coagulate, and cut, so after that we will have the completely dissected insular surface. After dissection of these branches, we expose the circular sulcus of the insula and we proceed to the dissection and resection of the insula exactly in the white matter just under the cortex of the insula. If we stay perfectly in the white matter there will be no bleeding and at the end the insula can be totally resected, giving this aspect as in this specimen in the right-hand side.

### 4:46 Corpus Callosotomy.

The next step is the exposure of the lateral ventricle for callosotomy, and we start from anterior to posterior. We start with the section of the horizontal fibers in the frontal region exposing the skull base and the sphenoid and with subpial dissection until we find the olfactory nerve, which is the landmark for the exposure of the gyrus rectus (as you can see here in this arrow) and the midline. After this, we expose completely the lateral ventricle and, in the midline, we proceed to the callosotomy by exposing the anterior cerebral artery. The artery is followed anteriorly all the way in the direction of the midline that we have already exposed in the frontal base. So, you can see how to find the cerebral artery anteriorly until the midline is exposed and, posteriorly, we do the same in the direction of the splenium; anterior exposure of the midline as shown in these arrows and posteriorly until we find the blue line of the splenium that represents the vein of Galen. We take extreme precaution not to make any kind of lesion of this vein.

### 7:18 Mesial Temporal Disconnection and Final Considerations.

The next step is the exposure of the temporal horn completely, and in the anterior direction you follow the hippocampus until the exposure of the amygdala. The amygdala is resected until we see the free edge of the tentorium. In the posterior direction we expose completely the hippocampus and progressively the hippocampal tail, and we find the fornix and the fimbria which will be sectioned. By doing this the temporal horn is completely disconnected. The resection of the temporal lobe is performed only when indicated—for instance, in cases of hemimegalencephaly. You see the body of the hippocampus here, the tail, as you can see by this arrowhead. Comparing with the specimen on the right side in the same position, you see the tail of the hippocampus and the fimbria-fornix. In children, the most common surgery that we perform in epilepsy centers is hemispherotomy now. This is completely different from the adults where the temporal lobectomy continues to be the first procedure. There are difficult cases, for instance, in cases of Sturge-Weber with leptomeningeal angiomatosis, but what is dangerous in these cases, and in hemimegalencephaly cases, are the veins that can be distorted and dilated, and we may have ischemic problems or even hemorrhage for deeply seated veins. The other cases are hemispheric polymicrogyria, mainly the perisylvian polymicrogyria that can be difficult for the neurosurgeon. So, in conclusion, hemispheric epilepsy is frequent in children and catastrophic progression is common. Surgery should be performed as early as possible. Hemispherotomy is the procedure of choice, although good experiences have been reported with anatomical hemispherectomy in selected cases. The choice of the technique should take in consideration the experience of the surgeon, the anatomy, the etiology, and the age of the patient. Thank you very much.

## Acknowledgments

We would like to thank Jose Hermes do Prado, MD, for his invaluable help in the preparation and editing of the video archive.

## Disclosures

The authors report no conflict of interest concerning the materials or methods used in this study or the findings specified in this publication.

## Author Contributions

Primary surgeon: Machado. Assistant surgeon: Volpon Santos. Editing and drafting the video and abstract: both authors. Critically revising the work: both authors. Reviewed submitted version of the work: both authors. Approved the final version of the work on behalf of both authors: Volpon Santos. Supervision: Machado.

## Supplemental Information

### Patient Informed Consent

As a retrospective video which did not include exposure of any personal details of patients, patient informed consent was not applicable to this study.

## References

[1] Rasmussen T Hemispherectomy for seizures revisited Can J Neurol Sci 1983 10 2 71 78 6861011 10.1017/s0317167100044668

[2] Villemure JG Rasmussen T Functional hemispherectomy: methodology J Epilepsy 1990 3 Suppl 177 182

[3] Delalande O Pinard JM Basdevant C Hemispherotomy: a new procedure for central disconnection Epilepsia 1992 33 3 Suppl 99 100

[4] Villemure JG Mascott CR Hemispherotomy: the periinsular approach Technical aspects. Epilepsia 1993 34 Suppl 6 48

[5] Villemure JG Mascott CR Peri-insular hemispherotomy: surgical principles and anatomy Neurosurgery 1995 37 5 975 981 8559348 10.1227/00006123-199511000-00018

[6] Shimizu H Maehara T Modification of peri-insular hemispherotomy and surgical results Neurosurgery 2000 47 2 367 373 10942009 10.1097/00006123-200008000-00018

